# Revisiting Radiotherapy for Hidradenitis Suppurativa: Clinical Outcomes, Safety, and Optimization Strategies: A Systematic Review

**DOI:** 10.3390/jcm15083164

**Published:** 2026-04-21

**Authors:** Michal Poltorak, Pawel Banatkiewicz, Lukasz Poltorak, Maciej Szwast, Irena Walecka

**Affiliations:** 1 The National Institute of Medicine of the Ministry of the Interior and Administration, Woloska 137, 02-507 Warsaw, Poland; pawel.banatkiewicz@pimmswia.gov.pl (P.B.); irena.walecka@pimmswia.gov.pl (I.W.); 2 Electrochemistry@Soft Interfaces Team, Department of Inorganic and Analytical Chemistry, Faculty of Chemistry, University of Lodz, Tamka 12, 91-403 Lodz, Poland; lukasz.poltorak@chemia.uni.lodz.pl; 3 Department of Chemical and Process Engineering, Warsaw University of Technology, Warynskiego 1, 00-645 Warsaw, Poland; maciej.szwast@pw.edu.pl; 4 Department of Dermatology, Centre of Postgraduate Medical Education, 02-507 Warsaw, Poland

**Keywords:** 3D printing, boluses, applicators, radiotherapy, hidradenitis suppurativa, chronic inflammatory skin disease

## Abstract

**Objectives**: Hidradenitis suppurativa (HS) is a chronic, inflammatory skin disease that significantly impairs patients’ quality of life, especially in its moderate to severe forms. Traditional treatments, including antibiotics, hormonal therapies, and surgery, often fail to provide long-term relief in such cases. This study aims to explore the role of radiotherapy, particularly with the use of 3D printing technology to create personalized boluses and applicators, as an adjunctive treatment for refractory HS. A systematic review of published studies was conducted to assess the efficacy of radiotherapy in managing HS, with a specific focus on studies using 3D printing technology to create customized boluses and applicators. **Methods**: Publications from databases such as PubMed, Scopus, and Google Scholar were analyzed for studies detailing radiotherapy techniques, dosing regimens, and the use of 3D-printed devices in HS treatment. The studies selected included those employing both external beam radiotherapy and brachytherapy, with particular emphasis on patient outcomes and adverse effects. **Results**: The reviewed studies highlighted a growing body of evidence supporting the use of radiotherapy for HS, especially in severe or treatment-resistant cases. The use of 3D-printed boluses and applicators in radiotherapy demonstrated significant improvements in treatment precision and patient comfort. Personalized treatment plans allowed for more accurate dose distribution, minimized air gaps, and reduced exposure of healthy tissue. No major long-term toxicity was reported across the majority of studies. **Conclusions**: Radiotherapy, particularly when combined with 3D printing technology, presents a promising treatment option for patients with severe or refractory HS. Customizable boluses and applicators enhance the precision of radiotherapy by conforming to irregular skin surfaces, thereby improving dose conformity and reducing side effects. Despite the positive results, further research is needed to assess the long-term safety and clinical feasibility of this approach. The integration of 3D printing in radiotherapy could significantly improve treatment outcomes, offering a more personalized and effective therapeutic option for HS patients.

## 1. Introduction

Hidradenitis suppurativa (HS) is a chronic, recurrent, and debilitating inflammatory dermatosis characterized by painful nodules, abscesses, sinus tracts, and scarring, predominantly affecting intertriginous areas, such as the axillae, groin, and inframammary regions [1,2,3,4,5,6,7,8]. The disease imposes a substantial physical, psychological, and social burden, significantly impairing patients’ quality of life [9]. Current management strategies for HS encompass a broad spectrum of interventions, including lifestyle modifications, topical and systemic antibiotics, hormonal therapies, biologic agents targeting inflammatory pathways, and procedural modalities ranging from incision and drainage to wide local excision [5,7,10]. Despite the availability of these therapies, many patients, particularly those with moderate to severe disease, experience inadequate responses. Treatment may also be limited by comorbidities, potential drug interactions, adverse effects, or contraindications to surgery, further complicating disease management [11,12,13]. Even combination therapies, commonly employed to maximize efficacy, often fail to achieve sustained remission, highlighting the significant unmet clinical need for alternative or adjunctive treatment approaches that are both effective and safe across diverse patient populations [14,15].

Radiation therapy is a widely used therapeutic modality that employs high energy electromagnetic waves or charged particles to induce deoxyribonucleic acid (DNA) damage in target cells, leading to cell death or growth arrest [13,14,15]. It can be delivered externally (external beam radiotherapy) or internally with contact using radioactive sources (brachytherapy), allowing precise targeting of diseased tissues while minimizing exposure to surrounding healthy structures. Modern techniques enable highly conformal dose delivery, reducing toxicity and improving treatment efficacy, and radiation can be applied with curative, adjuvant, or palliative intent depending on the clinical context [16,17,18]. Radiation therapy is not limited to oncologic indications. It is also employed in certain benign conditions, such as vascular malformations [19], keloids [20], or hyperproliferative disorders [21], where localized tissue control is desired. Treatment planning relies on advanced imaging and dosimetric calculations to optimize therapeutic effect while preserving normal tissue function [22,23]. Continuous technological advancements, including intensity modulated radiotherapy (IMRT) [24], volumetric modulated arc therapy (VMAT) [25], high-dose rate (HDR) brachytherapy [26], and surface-guided radiation therapy (SGRT) [27], have significantly enhanced the precision, safety and versatility of radiation therapy in modern clinical practice. A key focus in contemporary radiation therapy is treatment individualization, which aims to adapt therapy to the specific anatomy and pathology of each patient. One emerging approach is the use of 3D printing, for example, to fabricate patient-specific applicators for HDR brachytherapy or to create individualized boluses from suitable materials for external beam therapy. Although these technologies show great promise in improving dose conformity and sparing healthy tissue, their widespread clinical implementation remains limited and under active development.

Currently, the literature summarizing the evidence regarding the use of radiotherapy in HS is limited [28]. In this work, we systematically review the available studies on the efficacy of radiotherapy for patients with HS and compare it with current standard treatment modalities, including lifestyle interventions, antibiotics, hormonal therapies, biologics, and procedural approaches. Furthermore, we aim to explore and refine treatment paradigms by identifying the most appropriate radiotherapy modality whether external beam (teletherapy) or internal/contact (brachytherapy) and determining optimal dosing strategies. A key objective is to individualize therapy for each patient, leveraging advanced technologies such as 3D printing to create patient-specific applicators or boluses, thereby enhancing treatment precision, maximizing efficacy, and ensuring patient safety and comfort. By integrating these approaches, we hope to provide a framework for optimizing radiotherapy as a viable, tailored, and complementary treatment option for HS.

## 2. Methods

### 2.1. Search Strategy and Study Design

This systematic review was conducted in accordance with the Preferred Reporting Items for Systematic Reviews and Meta Analyses (PRISMA 2020) guidelines (see Table S1). A comprehensive literature search was performed across three electronic databases: PubMed, Scopus, and Google Scholar. The search strategy combined controlled vocabulary (MeSH terms, where applicable) and free text keywords related to HS and radiotherapy.

The following search terms were used: (“hidradenitis suppurativa” OR “acne inversa”) AND (“radiotherapy” OR “radiation therapy” OR “low dose radiotherapy”) AND (“3D printing” OR “three dimensional” OR “custom bolus” OR “patient specific bolus”). In addition, the reference lists of all included studies were manually screened to identify further relevant publications not captured in the initial search.

This systematic review was not registered in a publicly accessible database. The literature search was conducted up to January 2026.

### 2.2. Eligibility Criteria

Studies were considered eligible if they met the following criteria:

(i) Original research articles, including case reports, case series, and observational studies;
(ii) Studies evaluating the use of radiotherapy in patients with HS;
(iii) Studies reporting clinical outcomes, treatment parameters, or safety data;
(iv) Studies describing either external beam radiotherapy or brachytherapy, with or without the use of 3D-printed devices.

Exclusion criteria included the following:

(i) Review articles, editorials, and conference abstracts without full data;
(ii) Studies not involving HS;
(iii) Publications lacking sufficient methodological or clinical detail.

### 2.3. Study Selection Process

All records identified through database searching were imported into a reference management system, and duplicates were removed. The study selection process was conducted in two stages.

In the first stage, titles and abstracts were independently screened by two reviewers to assess potential eligibility. In the second stage, full-text articles of potentially relevant studies were retrieved and evaluated against the predefined inclusion and exclusion criteria.

**Figure 1:**
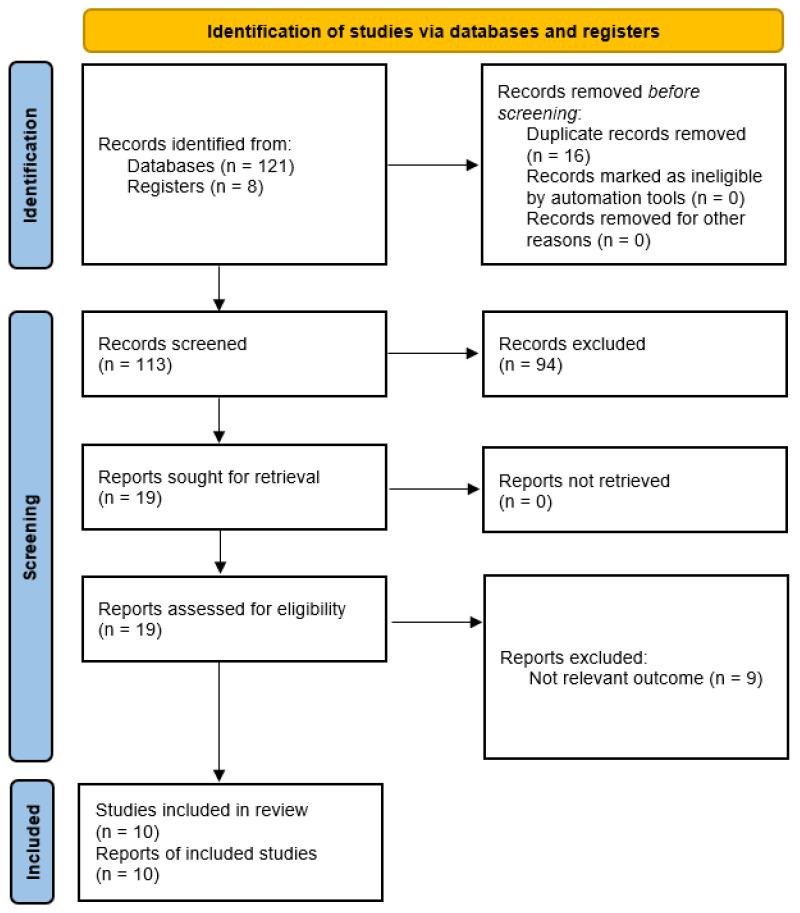
Illustrating the study selection process.

Any discrepancies between reviewers were resolved through discussion, and when necessary, consultation with a third reviewer. Reasons for exclusion at the full-text stage were documented to ensure transparency. The overall study selection process is summarized in the PRISMA flow diagram (Figure 1).

### 2.4. Data Extraction

Data extraction was performed independently by two reviewers using a standardized data collection form. Extracted data included the following:

- Study characteristics (author, year, study design);
- Number of patients;
- Anatomical location of lesions;
- Radiotherapy modality and parameters (dose, fractionation);
- Prior treatments;
- Clinical outcomes;
- Reported adverse effects.

Disagreements were resolved by consensus.

### 2.5. Quality Assessment and Risk of Bias

Given that the available evidence consisted predominantly of case reports and small case series, methodological quality and risk of bias were assessed using the Joanna Briggs Institute (JBI) critical appraisal tools appropriate for each study design.

Each study was evaluated across relevant domains, including patient selection, clarity of intervention description, outcome reporting, and follow-up adequacy. Based on these criteria, studies were qualitatively classified as having low, moderate, or high risk of bias.

Due to the heterogeneity of study designs, outcome measures, and small sample sizes, a quantitative meta-analysis was not feasible. Instead, a narrative synthesis of the evidence was performed, with particular attention to differences in study design and level of evidence.

### 2.6. Data Synthesis

Given the limited and heterogeneous nature of the available literature, results were synthesized qualitatively. Studies were grouped according to their design and level of evidence, including case reports, case series, and modeling or dosimetric studies. This approach allowed for a structured interpretation of findings while accounting for differences in methodological rigor.

This approach aimed to provide a structured and transparent selection of relevant studies. The available evidence is limited to case reports and small case series, precluding quantitative synthesis. Nevertheless, the application of a PRISMA compliant search strategy helped minimize selection bias.

The study selection process is presented in Figure 1 (PRISMA flow diagram).

## 3. Advantages of 3D-Printed Boluses and Applicators in Radiotherapy

HS presents anatomical and clinical challenges that make precise radiotherapy delivery difficult. Three-dimensional (3D) printing technology enables the creation of individualized devices printed boluses for teleradiotherapy and printed applicators for brachytherapy that significantly enhance treatment precision, comfort, and outcomes [29,30,31].

In teleradiotherapy, achieving consistent surface dose is essential, yet conventional boluses often fail to conform to areas affected by HS, such as the axillae, groin, or inframammary folds. Printed boluses are designed from patient imaging, reproducing complex contours, minimizing air gaps, and improving dose homogeneity [32]. Additionally, by bringing the dose closer to the skin surface, the bolus allows effective irradiation of larger and more superficial treatment fields, which is particularly useful in extensive HS involvement. Their patient-specific design supports reproducibility, reduces setup variability, and enhances quality assurance. Lightweight, comfortable materials improve compliance and reduce motion, while variable thickness allows for optimization of surface dose [29,30,31]. Digital planning and rapid fabrication streamline workflow and reduce manual labor.

Brachytherapy is valuable for localized or postoperative HS lesions, offering high conformity and rapid dose fall off. However, traditional applicators lack adaptability to irregular anatomy. Printed applicators can be customized to match surgical sites and complex body regions, enabling optimal source geometry and sparing of surrounding tissue [26,33]. Dedicated channels based on treatment planning improve dose accuracy and stability. Ergonomic designs enhance comfort and reduce displacement risk [33]. Integration with computed tomography (CT)/magnetic resonance imaging (MRI) facilitates precise dose calculation and efficient setup, shortening procedure time [34].

Both printed boluses and applicators provide key advantages: personalization, precision, and comfort. They improve treatment quality, reduce toxicity, and support consistency across sessions [29,30,31]. By enabling treatment in previously challenging regions, these devices advance individualized radiotherapy in HS and may reduce recurrence and radiation-related side effects. Although initial investment is required, long-term benefits include improved outcomes and workflow efficiency.

Future developments in flexible, biocompatible materials and AI-assisted design will further enhance customization. Point of care printing may allow same day production, improving accessibility. Ongoing collaboration between clinicians and engineers will drive innovation and standardization [29,30].

Printed boluses and applicators represent a major advancement in the radiotherapeutic management of HS. Their ability to deliver personalized, precise, and comfortable treatment improves the feasibility and effectiveness of both teleradiotherapy and brachytherapy, with the potential to enhance patient outcomes and broaden therapeutic options.

## 4. Conventional Medical and Surgical Treatments for HS

Traditional management of HS has long relied on methods aimed at reducing inflammation, preventing secondary infections, and minimizing recurrence. Before the introduction of biologic agents, treatment strategies were primarily based on topical, systemic, hormonal, and surgical interventions. Despite therapeutic advances, these traditional approaches remain essential, particularly in patients with mild or moderate disease where accessibility, safety, and cost effectiveness are key factors.

**(i)**  **Keratolytic and Antiseptic Topical Therapy in HS**

Topical therapy continues to play an important role, especially in early-stage HS. Among topical agents, resorcinol remains one of the most established and effective treatments. Its keratolytic and antiseptic properties promote follicular desquamation, accelerate lesion healing, and alleviate pain and itching. The cream is usually applied once daily, with twice daily use recommended during exacerbations. Adverse reactions are rare and typically limited to mild local irritation or contact dermatitis; systemic absorption is negligible [35,36,37].

**(ii)**  **Clindamycin as a Topical Treatment for HS**

Another first-line topical agent is clindamycin in gel or solution form, the only antibiotic with proven efficacy in randomized clinical studies. It is particularly effective in reducing superficial inflammatory lesions and abscesses, although less so in deep nodules and sinus tracts. Prolonged use may lead to bacterial resistance or local irritation; however, it remains a cornerstone of topical HS therapy, either alone or in combination with systemic antibiotics [7,38].

**(iii)**  **Systemic Antibiotics for HS: Doxycycline and Minocycline**

When topical measures are insufficient, systemic antibiotic therapy becomes the next step. Tetracyclines, such as doxycycline and minocycline, are widely used as first-line systemic options due to their strong anti-inflammatory effects [7,39]. They suppress neutrophil chemotaxis and cytokine activity, leading to reduced pain and lesion count. Clinical studies have shown response rates exceeding 60% after several weeks of treatment [40]. These agents are well tolerated, although gastrointestinal upset, photosensitivity, or hepatotoxicity may occur.

**(iv)**  **Systemic Combination Therapy in HS Unresponsive to Tetracyclines**

In more extensive or refractory disease, combination therapy with clindamycin and rifampicin provides significant clinical benefit. This regimen is particularly useful for patients who fail to respond to tetracyclines. Evidence indicates marked improvement and, in some cases, complete remission after several weeks of therapy [41,42,43]. However, its use requires caution, as clindamycin may induce Clostridioides difficile colitis, and rifampicin can interfere with hepatic metabolism, reducing the effectiveness of other medications [7].

**(v)**  **Intralesional Corticosteroids for Acute Flares in HS**

In acute inflammatory flares, intralesional corticosteroids, most commonly triamcinolone acetonide, can provide rapid symptom relief. Improvement is often seen within 24 h, and complete resolution typically occurs within a few days [44,45]. These injections are especially valuable for isolated, painful nodules and can significantly reduce patient discomfort during exacerbations [7].

**(vi)**  **Retinoid Therapy for Early or Follicular HS**

For selected cases, systemic retinoids such as acitretin or etretinate offer an alternative approach, particularly in early or follicular forms of the disease. By normalizing keratinocyte differentiation and reducing follicular occlusion, they help prevent new lesions and diminish inflammation. Multiple clinical reports confirm meaningful improvement and reduced recurrence rates in a considerable proportion of patients [7,46,47].

**(vii)**  **Hormonal Therapy in HS**

Hormonal therapy may also be beneficial, particularly in women with hyperandrogenism or polycystic ovary syndrome. Combined oral contraceptives containing cyproterone acetate and ethinylestradiol lower androgen levels, decrease sebaceous activity, and reduce follicular occlusion. This can lessen flare frequency and improve overall disease control [7,48,49]; however, potential hepatic and thromboembolic risks require individualized assessment.

**(viii)**  **Surgical Management of HS**

Surgery is indicated when disease is rather inactive, recurrent, scarring, or field based. The choice of procedure is guided by lesion pattern, anatomical site, and patient priorities (function, downtime, cosmesis) [50,51,52,53,54,55]. For acute, fluctuant abscesses, incision and drainage provides rapid decompression and analgesia but is not definitive and carries very high relapse rates. Thus, it should be used for urgent control rather than long-term management [5,56,57]. For limited tract predominant disease, deroofing/laying open removes the sinus roof while preserving uninvolved tissue; it is frequently office based and yields favorable function and cosmesis, with prospective series reporting low teen recurrence rates [58,59,60]. In more extensive tunnel networks, STEEP (skin tissue-sparing electrosurgical peeling) offers a structured, tissue conserving excision strategy, with reported recurrence of around 29% in retrospective cohorts, reflecting case mix and technique standardization [61]. CO_2_ laser allows focal vaporization of nodules, abscesses, and fistulous tracts while maximizing tissue preservation [62,63]. For field disease, wide local excision remains the gold standard; margins are individualized to clinical spread and followed by planned reconstruction [64,65,66].

**(ix)**  **Reconstruction Strategies and the Role of Radiotherapy in HS Management**

Post excisional reconstruction spans primary or delayed primary closure, secondary intention healing, split thickness skin grafts, or local/regional flaps. Selection should balance time to heal, function, scar quality, and recurrence risk and be planned at the index operation [64,65,66]. Practical optimization includes smoking cessation, weight control, hygiene, and targeted infection management. Patients should be counselled that tissue-sparing techniques aim to reduce morbidity, while wide excision offers the most durable field control. In addition, patients should be advised that recurrence varies by technique and closure method [5,56,57,59,60,61,62,63,64,65,66]. In addition to established therapeutic options, radiotherapy has recently gained renewed interest as a potential modality in HS management. It may be applied as teletherapy or brachytherapy, depending on lesion depth and localization. With modern individualized approaches, such as the creation of customized boluses or applicators using 3D printing technology, treatment precision and tissue conformity can be significantly enhanced. This personalized planning allows for optimal dose distribution and reduced toxicity, providing a valuable alternative in selected refractory cases. The methodology and outcomes of these techniques are discussed in greater detail in the subsequent section.

The present review is based on the “Polish guidelines for the diagnosis and treatment of hidradenitis suppurativa” [67] developed by national experts in the field, which remain a key reference for evidence based clinical practice in HS management. These guidelines emphasize the continued importance of traditional, multidisciplinary treatment approaches, even in the era of biologic therapy, as they form the foundation upon which modern, individualized care continues to evolve.

## 5. Clinical Reports on HS with the Use of Radiotherapy

In this report, we present a review of the available literature on the use of radiotherapy in the treatment of HS. Although radiotherapy is not considered a standard therapeutic approach for this condition, an increasing number of studies suggest its potential benefit in patients with severe or treatment-resistant disease. It must be stated that radiotherapy for HS treatment is applied as the last resort and can be introduced when other treatment methodologies fail. This report summarizes data from 10 published studies, focusing on the efficacy, safety, and optimal radiotherapy regimens applied in HS management, as well as highlighting current evidence gaps and potential future research directions. These studies represent the final set of articles included after full-text eligibility assessment. A detailed summary of the reviewed studies is presented below in Table 1.

The clinical data presented in Table 1 should be interpreted with caution. Most of the included studies are based on small patient numbers, retrospective designs, and relatively short follow-up periods, which limit the generalizability and strength of the conclusions. In addition, outcome measures are often non-standardized and heterogeneous, making direct comparisons between studies difficult.

## 6. Risk of Squamous Cell Carcinoma in Chronic HS Lesions

Cutaneous squamous cell carcinoma (cSCC) may represent a rare yet exceptionally aggressive complication of long standing HS. It most frequently develops in the gluteal–perianal and perineal regions, where chronic inflammation, fistula formation, and scarring persist for many years. Literature reports indicate that this malignancy occurs more commonly in men with a long-standing, scar-forming course of HS, and its diagnosis is often delayed, which significantly worsens the prognosis [78,79]. In a classical clinicopathological series describing consecutive cases of cSCC arising in the context of HS, the authors emphasized the predominance of gluteal perianal localization, the long duration of HS before malignant transformation, and the increased risk of metastasis [80]. The mechanisms underlying this transformation include chronic inflammation, continuous tissue damage, fistula formation, and recurrent bacterial infections. In recent years, several systematic reviews have analyzed reported cases of cSCC developing within HS lesions [77,79,80,81], gathering dozens of case descriptions worldwide. These studies highlight several key features: (i) the prolonged duration of HS prior to malignant transformation; (ii) the predominance of anogenital localization; (iii) the need for high oncological vigilance including biopsy of chronic, non-healing, or atypical wounds; and (iv) the role of wide margin surgical excision as the cornerstone of treatment. In advanced cases, radiotherapy and systemic therapy should be considered according to the established standards for high risk cSCC management [79]. The prognosis for these patients remains poor. Clinical series involving perineal, gluteal, and perianal localizations have shown high rates of regional and distant metastases. Therefore, an aggressive diagnostic approach is recommended, including early biopsy of all chronic or atypical HS lesions, prompt imaging for staging, and combined modality treatment. Surgical excision remains the gold standard, often extensive, while radiotherapy may be used in inoperable or borderline cases, supported by systemic therapy [78]. From a practical standpoint, in patients with HS, particularly men with a long disease duration and anogenital involvement, a low threshold for biopsy of any verrucous, over granulating, or bleeding lesion should be maintained. Regular dermatologic follow-up and close multidisciplinary collaboration between dermatologists, surgeons, and oncologists are essential for early detection of malignant transformation and improvement of outcomes [79].

Given the aggressive nature of cSCC arising in HS, this issue warrants deeper investigation, especially regarding the optimization of adjuvant treatment modalities, such as radiotherapy. One promising direction involves the implementation of 3D printing technology for patient-specific design of boluses or applicators, allowing for adjustment of their thickness and shape according to lesion location and disease stage. This approach enables more precise surface contouring, improved dose distribution, and reduced irradiation of surrounding healthy tissues. The key to therapeutic success, however, lies in the appropriate selection of total dose and fractionation, tailored to the lesion’s depth, anatomical region, and patient condition. Integrating 3D printing technology into radiotherapy planning could, therefore, significantly enhance the efficacy and safety of treatment for this challenging clinical complication of HS.

Radiation-induced carcinogenesis should also be considered, as malignant transformation may, in rare cases, represent a late complication of radiotherapy. Furthermore, during the course of HS treatment, various oncological complications may occur and should be taken into account in long-term patient management. However, a detailed discussion of these mechanisms and risks remains beyond the scope of the present work.

## 7. Guidelines for Anticipated Application of 3D Printing in HS Management

Several factors need to be considered when planning radiotherapy for HS or general HS treatment: (i) the type of 3D printing technology to be used; (ii) the cost of the hardware; (iii) the user friendliness of the chosen technology; (iv) the availability of suitable printable materials for the intended applications; (v) the time required for fabricating a single print; and (vi) the scalability of bolus or applicator.

To date, the application of 3D printing in HS treatment has been limited to a few notable examples, where additively manufactured objects served as carriers for antibiotics applied over affected skin regions. Aliyazdi et al. printed a polycaprolactone scaffold filled with a collagen matrix, providing support for human hair follicles. The introduction of Staphylococcus aureus (a known contributor to HS) led to follicular infection, which was then treated with lipid nanoparticles loaded with rifampicin or rifampicin alone. The findings demonstrated that the 3D printable model deigned with channels supplying nutrients to the follicles could be used to evaluate a treatment protocol before it is applied to patients [82]. Another example by Gezek et al. involved 3D printing an open PLA-based scaffold, which was subsequently covered with rifampicin-loaded PLA particles using an electrohydrodynamic atomization process. It was shown that the printed and modified objects exhibited activity against Staphylococcus aureus and Escherichia coli, as well as enabling controllable and programmed drug release [83]. These two studies, however, do not address the broader topic of pharmaceutical treatment for HS and its integration with 3D printing technology.

Technologies such as direct ink writing (DIW) could be utilized to create hydrogel based dressings, with predefined drug concentrations loaded into the ink during the formulation stage. We also see significant potential in combining radiotherapy and pharmaceutical approaches, both following the principles of personalized medicine. Simple body scanning can provide information about the often complex body architecture within HS lesions, which could then be converted into solid applications for radiotherapy, as well as hydrogel-based, drug-loaded printed wound dressings.

Currently, the most commonly used 3D printing technology for fabricating boluses and applicators in radiotherapy is fused deposition modeling (FDM) (refer to [29]), while digital light processing (DLP) and stereolithography (SLA) remain less commonly applied. However, we are confident that the increasing availability of biocompatible, medical grade resins [84,85,86] will soon lead to the development of objects capable of printing complex skin geometries for controlled drug release, as well as the fabrication of applicators and boluses.

## 8. Future Perspectives

Radiotherapy has reemerged as a valuable therapeutic option for patients with severe or treatment resistant HS, providing durable symptom control and good tolerability in appropriately selected cases. Despite its clinical potential, the delivery of radiotherapy for HS remains technically challenging due to the irregular and anatomically complex surfaces of affected regions, such as the axillae, groins, or perineal folds. In recent years, the rapid development of three-dimensional (3D) printing technologies has opened new possibilities for improving precision, comfort, and personalization in radiotherapy, mainly in the treatment of various types of skin cancer. One of the most promising uses of 3D printing in this context is the creation of patient-specific boluses. Traditional flat bolus materials often fail to achieve uniform skin contact, especially over concave or folded regions, leading to air gaps and dose inhomogeneity—challenges that are encountered during HS treatment. By using patient CT data or simple 3D surface scans originating from a laser scanner, 3D-printed boluses can be fabricated to match the exact contour of the skin, ensuring accurate dose buildup and homogeneous distribution. Clinical and dosimetric studies have confirmed that custom 3D-printed boluses significantly improve treatment accuracy compared to conventional materials, reducing air gaps and improving reproducibility across sessions [87,88,89]. Beyond bolus fabrication, 3D printing allows for the production of personalized shields and immobilization devices, particularly valuable when treating lesions near radiosensitive structures, such as the breast, genital, or perineal areas. Printed boluses/applicators made from biocompatible, high-density polymers have demonstrated excellent attenuation characteristics and mechanical stability, providing effective protection of adjacent organs at risk while maintaining patient comfort [88,90,91]. Furthermore, the integration of 3D printing into modern image guided and surface guided radiotherapy (IGRT/SGRT) workflows enhances the precision of superficial treatments. The ability to combine custom printed boluses with optical surface monitoring or cone beam CT verification has improved dose conformity and positioning accuracy, particularly for irregularly shaped targets or lesions in motion prone regions [89,92]. Recent clinical research has also focused on dosimetric validation and patient safety of 3D-printed boluses. One study demonstrated that the use of individualized boluses in adjuvant intensity modulated radiotherapy following radical mastectomy achieved excellent skin adherence, minimized hot spots, and reduced acute radiation dermatitis compared to standard materials [90]. Similarly, other validation studies confirmed high geometric accuracy and favorable dosimetric performance, supporting clinical feasibility in complex anatomical areas [93,94]. Such findings, while derived from oncologic indications, strongly support the potential transfer of this technology to inflammatory dermatoses such as HS, where surface irregularities are comparable. Looking ahead, future research should aim to evaluate the clinical feasibility of 3D-printed devices in HS radiotherapy through prospective dosimetric and patient reported outcome studies. To do this we proposed several guidelines along with challenges that should be overcome:

(i) Investigations into sterilizable and skin compatible printing materials are crucial. Since the most effective sterilization method involves high temperature and pressure (autoclaving), most thermoplastic materials, and thus FDM or FFF, are not suitable for the intended application. UV irradiation can also be effective, but it requires additional effort to ensure that the object is evenly exposed to irradiation from all angles. In this context, photocurable resins appear to be a better option, as they offer higher resistance to deformation at elevated temperatures and benefit from the added exposure to UV light, ensuring complete crosslinking.
(ii) Given the high probability of occupying large surface areas, HS treatment may require boluses with varying surface areas. In this regard, significant printing speeds are needed, and once again, DLP technology proves to be advantageous. The ideal scenario would involve scanning the patient’s body, 3D printing the object, and initiating the first treatment all during a single visit.
(iii) The scan of the body surface topography, converted into an.stl file, can be used to generate 3D-printed applicators/boluses for radiotherapy. These objects can exhibit an even surface topography while simultaneously delivering pharmaceuticals in a controlled manner.
(iv) The use of artificial intelligence (AI) based automatic segmentation to accelerate the modeling process is also warranted. AI is expected to be valuable in controlling the positioning of the bolus on the patient’s body and is anticipated to rapidly monitor changes in skin surface topography during treatment by analyzing surface scans and evaluating the treatment progress.

The integration of 3D printing with individualized radiotherapy planning could lead to a new paradigm of personalized, minimally invasive treatment for chronic inflammatory skin diseases such as HS.

## 9. Conclusions

HS is a challenging and debilitating condition that severely impacts patients’ quality of life. Despite advancements in treatment options, many patients continue to experience inadequate responses, particularly in moderate to severe cases. Radiotherapy, although not yet a standard treatment for HS, has shown promise in managing refractory or extensive forms of the disease. The ability of radiation therapy to deliver precise and localized treatment is particularly important in HS, where lesions affect anatomically complex and irregular regions such as the axillae, groin, and perineal folds. One of the most significant advancements in radiotherapy for HS is the integration of 3D printing technologies, which offer substantial improvements in treatment precision and personalization. By creating patient-specific boluses and applicators, 3D printing allows for better dose distribution, minimized air gaps, and improved treatment reproducibility. These custom-made devices enhance comfort, reduce motion, and optimize radiation delivery, particularly in challenging anatomical areas. While the theoretical advantages of 3D-printed boluses and applicators are established in oncologic dermatology, direct HS-specific clinical evidence remains limited. The extrapolation from other dermatologic and oncologic indications is reasonable, but we acknowledge that outcomes in HS may differ due to unique anatomical and inflammatory considerations. Practical implementation in HS requires careful attention to sterilization, regulatory compliance, material biocompatibility, and integration into clinical workflow. Future studies focusing on HS-specific applications will be essential to validate these anticipated benefits and to establish standardized protocols for routine clinical use. Despite the promising potential of 3D printing in radiotherapy for HS, there remain several challenges to overcome. These include developing suitable biocompatible materials for bolus fabrication, improving the speed and scalability of the printing process, and addressing sterilization concerns for clinical applications. Nevertheless, with continued advancements in 3D printing technology, the integration of artificial intelligence for treatment planning, and the potential for personalized therapeutic strategies, the future of radiotherapy in HS management looks promising. This review highlights the need for further research to assess the clinical feasibility and long-term outcomes of radiotherapy, particularly in combination with 3D printing, in the treatment of HS. By optimizing treatment paradigms and incorporating individualized approaches, we hope to provide a more effective and tailored solution for patients suffering from this challenging and often treatment resistant condition. The incorporation of 3D-printed devices could revolutionize HS treatment, offering a safer, more comfortable, and highly effective alternative or adjunct to existing therapies, thus improving patient outcomes and overall quality of life.

This review has several limitations. The included studies were predominantly case reports and small case series, which limits the strength of the conclusions. The heterogeneity of study designs and outcome measures precluded quantitative synthesis. Additionally, potential publication bias cannot be excluded, as only published studies were considered.

## Figures and Tables

**Table 1:** Summary of published reports on the use of radiotherapy in hidradenitis suppurativa.

Reference/Year	No. of Patients	Location	Used Energy	Radiation Dose and Fractionation	Previous Treatment(s)	Treatment Outcomes	Adverse Effects/Comments
Garcia-Grande A et al.; [68], 2013	10	Axillae, groin, perineal, intermammary folds	N/A	20 Gy (4–5 Gy × 4–5 fr)	Failed antibiotics, surgery, infliximab	≥50% complete, ≥90% partial improvement	No major toxicity
Fernandes NC et al.; [69], 2013	20	Axillary area (five cases); inguinal and axillary regions (four); gluteal and perineal/perianal areas (four); axillary, gluteal, perineal, and inguinal regions (five); gluteal and perineal areas (two cases); gluteal; perineal	N/A	3–8 Gy; in chronic/recurrent cases ≈ 10 Gy (≥2 series)	Topical/systemic antibiotics, surgery	Good control in selected cases	No major toxicity
Paul S et al.; [70], 2016	1	Painful nodules involving much of his scalp, neck, face, chest, and groin	Radioactive source	Superficial brachytherapy—10 Gy/4 fx (2.5 Gy per fx)	Fluconazole and griseofulvin for presumed tinea; prolonged antibiotics (minocycline, ciprofloxacin); no improvement observed	Well tolerated; no acute toxicity or recurrence during 11-week follow-up	No toxicity
Jansen J.T.M. et al.; [71], 2005	Model study	Various anatomical regions	MV	1–7 Gy/fx (simulation)	N/A	Risk estimation model	No acute toxicity; very low long-term carcinogenic risk (~1–3/1000); risk minimal in older patients
Boer J.; [72], 2021	64	Axillae	kV	4–6 biweekly fractions of 0.75–1 Gy (total 6 Gy); chronic cases: +4 daily × 2 Gy (up to total 14 Gy)	Failed antibiotics, surgery	85% complete remission (34/40 sites); no side effects after 40 years of follow-up	No late adverse effects
Patel SH et al.; [73], 2013	5	Axillae, inguinal, perineal	MV	7.5 Gy (2.5 Gy × 3)	Topical and oral antibiotics, surgery, other therapies	No complete responses; 53% partial response.	Low secondary malignancy risk
Sakyanun P et al.; [74], 2022	1	Bilateral axillae	MV	7.5 Gy (2.5 Gy × 3)	Antibiotics, multiple excisions with drainage, no improvement	Marked improvement by end of radiotherapy; at 3-month follow-up, skin smooth, no pus or odor	No toxicity
Walker R et al.; [75], 2025	7	Groins, buttocks, axillae, perineum	MV	7.5 Gy (2.5 Gy × 3) 9 Gy (1.5 Gy × 6)	Antibiotics, immunosuppressive agents, biologics, retinoids, deroofing	16% complete response, 60% partial response, 24% no response; 75% showed pain reduction after radiotherapy	No toxicity
Ciudad-Blanco C et al.; [76], 2025	1	Predominantly buttocks and perianal area with SCC	N/A	N/A	Immunotherapy (pembrolizumab/cemiplimab)	After radiotherapy, symptoms improved and lesion size decreased; treatment continued with cemiplimab	Good tolerance
Mark T et al.; [77], 2010	1	Bilateral inguino femoral regions	MV	4.5 Gy (1.5 Gy × 3 fractions over 2 days) + 6 Gy (2 Gy × 3 fractions over 2 days) + 7.5 Gy (2.5 Gy × 3 fractions over 2 days)	Conservative therapy with hygiene counseling and antibiotics; later refractory disease treated with surgical resection of all affected sites	Complete lesion resolution within 2 weeks; no acute or chronic side effects observed	Good tolerance

## Data Availability

No new data were created or analyzed in this study. Data sharing is not applicable for this review article.

## Supplementary Materials

The following supporting information can be downloaded at: https://www.mdpi.com/article/10.3390/jcm15083164/s1, Table S1: PRISMA checklist. Reference [95] is cited in the Supplementary Materials.
